# Speech Segmentation and Cross-Situational Word Learning in Parallel

**DOI:** 10.1162/opmi_a_00095

**Published:** 2023-07-28

**Authors:** Rodrigo Dal Ben, Isabella Toselli Prequero, Débora de Hollanda Souza, Jessica F. Hay

**Affiliations:** Universidade Federal de São Carlos, São Carlos, São Paulo, Brazil; University of Tennessee, Knoxville, Knoxville, TN, USA

**Keywords:** statistical learning, speech segmentation, cross-situational word learning, word learning

## Abstract

Language learners track conditional probabilities to find words in continuous speech and to map words and objects across ambiguous contexts. It remains unclear, however, whether learners can leverage the structure of the linguistic input to do both tasks at the same time. To explore this question, we combined speech segmentation and cross-situational word learning into a single task. In Experiment 1, when adults (*N* = 60) simultaneously segmented continuous speech and mapped the newly segmented words to objects, they demonstrated better performance than when either task was performed alone. However, when the speech stream had conflicting statistics, participants were able to correctly map words to objects, but were at chance level on speech segmentation. In Experiment 2, we used a more sensitive speech segmentation measure to find that adults (*N* = 35), exposed to the same conflicting speech stream, correctly identified non-words as such, but were still unable to discriminate between words and part-words. Again, mapping was above chance. Our study suggests that learners can track multiple sources of statistical information to find and map words to objects in noisy environments. It also prompts questions on how to effectively measure the knowledge arising from these learning experiences.

## INTRODUCTION

Learning a new language requires mastering several complex tasks. Research has shown that language learners can use statistical cues from their linguistic environment to overcome some of these challenges. For instance, learners can track conditional probabilities between syllables to discover words from continuous speech and between words and objects to learn the meaning of novel words across ambiguous situations. The present study explores how tracking conditional probabilities in audiovisual input may help learners to solve both tasks simultaneously. We combine two well established statistical learning tasks—speech segmentation (e.g., Romberg & Saffran, 2010; Saffran et al., 1996) and cross-situational word learning (e.g., Smith & Yu, 2008; Yu & Smith, 2007)—into a single paradigm.

Faced with continuous speech and only a few words in isolation (∼10%; Brent & Siskind, 2001), one of the crucial challenges for language learners is to segment streams of words into discrete units. Conditional probabilities between syllables (i.e., transitional probabilities; Krogh et al., 2013; Romberg & Saffran, 2010; Saffran et al., 1996) provide one cue that aids segmentation (for evidence of other cues, see Hay & Saffran, 2012; Johnson et al., 2014). In natural speech, syllables that form words tend to have higher likelihood of co-occurrence (higher Transitional Probabilities, TPs) in comparison to syllables across word boundaries (Swingley, 1999; but see Yang, 2004), which provides a potential cue to segmentation. For instance, in the sequence pretty#baby the TP of pre to ty is greater than the TP of ty to ba, this difference in TP could signal a word boundary for learners (Saffran et al., 1996). There is now a vast empirical literature showing that language learners can track differences in TPs across syllable sequences to segment continuous speech into discrete words (for reviews see Cannistraci et al., 2019; Cunillera & Guilera, 2018; but see Black & Bergmann, 2017). The experimental task in these studies usually starts by familiarizing participants with a continuous speech stream in which TP is the main cue to word boundaries. For instance, some syllables *always* occur together (creating a word), *sometimes* occur together (creating a part-word or a low TP word), or *never* occur together (creating a non-word). Following familiarization, participants' preferences for words, part-words, or non-words are measured. By and large participants differentiate words from foils (part-words or non-words), suggesting that they successfully tracked TP information to find words in the continuous speech stream.

Phonotactic probability (PP), the conditional probability of a syllable occurring in a given position of a word from a given language (Vitevitch & Luce, 2004), is another statistical cue to word boundaries (Benitez & Saffran, 2021; Mattys & Jusczyk, 2001; Mattys et al., 1999). For instance, in the same sound sequence pretty#baby, the English PPs^1^ of the words pretty and baby are comparable (≈ 0.0440, ≈ 0.0050, respectively) and both are higher than the PP of the part-word ty#ba (≈ 0.0022), which could signal word boundaries to language learners. The combined information of TPs and PPs can promote—when both cues point to word boundaries—or impair speech segmentation—when they provide conflicting information about word boundaries. Evidence suggests that this happens when TP is combined with legal versus illegal PPs (Finn & Hudson Kam, 2008), with high versus low PPs (Mersad & Nazzi, 2011), and even with subtle differences in high PPs (Dal Ben et al., 2021). In previous work, we argued that careful consideration of phonotactics from participants’ natural languages should be an integral part of the stimuli design of statistical speech segmentation studies (Dal Ben et al., 2021). This is especially true when studying adults, who will promptly bring their extensive learning history and expectations from their natural languages’ PPs to the experimental task (Steber & Rossi, 2020; Sundara et al., 2022).

Assigning meaning to words is another challenge for language learners. There is evidence that, early in development, recently segmented words (with stronger TPs) are treated as better candidate labels on subsequent mapping tasks (Graf Estes et al., 2007; Hay et al., 2011). While the benefit of high TP sequences during word learning appears to diminish across development (Mirman et al., 2008; Shoaib et al., 2018), learners continue to be remarkably successful both at segmenting speech using TP information (Saffran et al., 1996; but see Black & Bergmann, 2017) and at making one-to-one mappings between labels and referents (Graf Estes, 2009; Graf Estes et al., 2007; Lany & Saffran, 2010). Furthermore, across the lifespan, language learners rely on phonotactics from their natural languages when learning novel words, with words with stronger PPs being learned faster and more accurately than words with weaker PPs (Graf Estes et al., 2011; Storkel et al., 2013; but see Cristia 2018). However, this might not be true when learning novel words in ambiguous situations (Dal Ben et al., 2022).

In everyday life, several words are presented with several potential referents at the same time, creating ambiguous learning experiences (Quine, 1960). A growing empirical literature shows that learners can track word-object co-occurrences across ambiguous situations to find the meaning of words (for a recent meta-analysis, see Dal Ben et al., 2019; but see Smith et al., 2014). The experimental task in these studies usually familiarizes participants with a series of ambiguous trials. On each trial, two (or more) words are presented with two (or more) objects. On any given trial, there is insufficient information to solve the ambiguity. However, if participants compare word-object conditional probabilities across trials, word-object relations can be learned^2^ (Smith & Yu, 2008; Yu & Smith, 2007).

The evidence that statistical information can promote both speech segmentation and cross-situational word learning prompts the question of whether these processes unfold in sequence or in parallel. Related evidence for the latter is reported by Cunillera, Laine, et al. (2010). Adults were familiarized with a continuous speech stream and, at the same time, with a stream of objects. When the first word was being played, its corresponding object was displayed on the screen; when the second word started, its corresponding object replaced the previous one, and so forth. From this dynamic presentation, participants were able to segment words from the continuous speech and to map them to its corresponding objects in parallel. In addition, in a follow-up study, François et al. (2017) replicated the findings and showed neurophysiological markers for online simultaneous speech segmentation and mapping. Although these studies have shown that segmentation and mapping can happen in parallel (see also Shukla et al., 2011 for a related task with infants), both used non-ambiguous word learning tasks.

Intuitively, adding mapping ambiguity could make the simultaneous task too challenging. However, Yurovsky et al. (2012) have shown that adults can simultaneously segment labels from phrases and map them to objects across ambiguous presentations. Using an adaptation of the cross-situational word learning paradigm (Yu & Smith, 2007), adults were exposed to scenes with two novel objects. On each trial, they would see only one object and hear a sentence that included a word labeling it among other function words. When the position and the onset of labels in the sentences matched the patterns of their natural language (i.e., final position, label preceded by a small set of words), participants were able to segment the labels and to map them to objects. Despite the additional demands that ambiguity might impose, the authors argued that the parallel solution of segmentation and mapping might happen in continuous iterations, as even partial speech segmentation would reduce mapping ambiguity and vice-versa (for similar evidence with multilingual adults see Tachakourt, 2023; for related evidence with other linguistic cues, see Feldman, Griffiths, et al., 2013; Feldman, Myers, et al., 2013). This is in line with proposals by Räsänen and Rasilo (2015). In a comprehensive combination of computational simulations and reanalyses of empirical data, the authors argue that tracking cross-modal conditional probabilities between words and objects in ambiguous situations may boost both speech perception and word learning, in comparison to tracking only TPs or word-object co-occurrences (for a similar argument, see Jones et al., 2010). Moreover, recent meta-analytic findings show that infants effectively integrate audio and visual information, from a variety of sources, when learning language (e.g., Cox et al., 2022; but see Frank et al., 2007, Johnson & Tyler, 2010, and Thiessen, 2010 for potential limits of this integration).

Here we further explore whether the integration of transitional probabilities, phonotactic probabilities, and word-object co-occurrences would promote speech segmentation and word learning across ambiguous presentations. Our study is guided by three main questions. First, we ask whether words can be segmented and mapped at the same time across dynamic ambiguous presentations. To answer this question, we adapted the design by Cunillera, Laine, et al. (2010) to combine a speech stream with several new objects in an ambiguous fashion. Second, we ask whether phonotactic properties of our stimuli would impact speech segmentation and cross-situational word learning in parallel. Answering this question allows us to better understand how multiple linguistic statistics can be combined when learning novel words across ambiguous situations (Saffran, 2020; Smith et al., 2018). Third, we ask whether this joint task would improve segmentation and mapping in comparison to separate tasks. To answer this question, we compared our current findings to data from our previous studies testing speech segmentation (Dal Ben et al., 2021) and cross-situational word learning (Dal Ben et al., 2022) separately, but using the same stimuli (same TP and phonotactic properties) and population.

## EXPERIMENT 1

To investigate whether words can be segmented and mapped simultaneously and whether differences in phonotactics would impact this joint performance, we exposed participants to continuous speech streams with varying distributions of phonotactics and TPs. At the same time, we also presented them with a series of objects, two at a time, that corresponded to the words in the speech streams. Critically, one of the languages had TPs and phonotactics aligned, consistently pointing to word boundaries. In another language, words and part-words had balanced phonotactics, with TPs being the only informative statistic to word boundaries. In a third language, TPs and phonotactics were in conflict: TPs pointed to word boundaries and phonotactic information pointed to syllables within-words (part-words).

To investigate whether the joint task would improve segmentation and mapping in comparison to separate tasks, we compared segmentation and mapping performance in the present combined task with performance in the individual tasks (i.e., speech segmentation only and cross-situational word learning only; Dal Ben et al., 2021, 2022, respectively).

### Method

#### Participants.

Sixty native Brazilian-Portuguese-speaking adults (*M*_*age*_ = 21.37 years, ± 3.27 *SD*, 32 female) participated. None of the participants reported any visual or auditory impairments that could interfere with the task. Participants were recruited online at the official Facebook group of Universidade Federal de São Carlos, where data was collected. They received no compensation for their in-person participation. The study was conducted according to the Declaration of Helsinki and the Ethics Committee of the host university approved the research (#1.484.847). Participants were randomly assigned to one of three groups.

### Stimuli and Design

#### Auditory Stimuli.

Three frequency-balanced languages from Dal Ben et al. (2021) were used (see Table 1). Each language contained six statistically defined disyllabic pseudo-words (TP = 1), which served as labels in our task. Test words and part-words in all Languages were frequency balanced (Aslin et al., 1998). In each language, half of the words were repeated 300 times (labeled H on Table 1) and the other half were repeated 150 times (labeled L on Table 1). The recombination of syllables from the words with higher frequency generated three part-words, used during test phase, that had lower TPs (TP = 0.5), but that were balanced in frequency with the test words (150 repetitions each; Aslin et al., 1998).

**Table 1.** Words and Part-words (grapheme and IPA) and their Phonotactic Probabilities (PP+ or PP−) and Frequency (High or Low) for the Balanced, and Aligned, Conflict Languages

**Language**	**Familiarization**				**Test**							
	**Words**		**PP**	**Freq**	**Words**		**PP**	**TP**	**Part-words**		**PP**	**TP**
Balanced	sute	[sute]	H+	H	nipe	[nipe]	H−	1.0	teba	[teba]	H−	0.5
	viko	[viko]	H+	H	tadi	[tad͡ʒi]	H−	1.0	kosu	[kosu]	H−	0.5
	bara	[baʁa]	H+	H	mide	[mide]	H−	1.0	ravi	[ʁavi]	H−	0.5
	nipe	[nipe]	H−	L								
	tadi	[tad͡ʒi]	H−	L								
	mide	[mide]	H−	L								
Aligned	dini	[d͡ʒini]	H+	H	sute	[sute]	H+	1.0	nipe	[nipe]	H−	0.5
	deta	[deta]	H+	H	viko	[viko]	H+	1.0	tadi	[tad͡ʒi]	H−	0.5
	pemi	[pemi]	H+	H	bara	[baʁa]	H+	1.0	mide	[mide]	H−	0.5
	sute	[sute]	H+	L								
	viko	[viko]	H+	L								
	bara	[baʁa]	H+	L								
Conflict	teba	[teba]	H−	H	nipe	[nipe]	H−	1.0	sute	[sute]	H+	0.5
	kosu	[kosu]	H−	H	tadi	[tad͡ʒi]	H−	1.0	viko	[viko]	H+	0.5
	ravi	[ʁavi]	H−	H	mide	[mide]	H−	1.0	bara	[baʁa]	H+	0.5
	nipe	[nipe]	H−	L								
	tadi	[tad͡ʒi]	H−	L								
	mide	[mide]	H−	L								

In addition, all words and part-words had legal and high phonotactic probabilities in Brazilian-Portuguese. Following previous research (Dal Ben et al., 2021), we decided to use only syllable sequences with high phonotactics (instead of legal vs. illegal or high vs. low; Finn & Hudson Kam, 2008; Mersad & Nazzi 2011) so that all syllable sequences would be phonotactically plausible in the participants’ native language. However, some syllable sequences had higher phonotactic probability than others (Table 1, PP+ or PP−). Phonotactics were calculated using Vitevitch and Luce’s (2004) algorithm and Estivalet and Meunier (2015) database of Brazilian-Portuguese biphones. Briefly, we divided the sum of the log (base 10) of token frequency of each biphone on each word position by the total log frequency of words with biphones in that given position (e.g., /mæ/ in the third biphone divided by the total log frequency of all words with at least three biphones). Then, using a custom search engine, we created six novel disyllabic words with consonant–vowel structure (CVCV) and with the highest possible phonotactic probability before becoming actual words in Brazilian-Portuguese (labeled PP+; Table 1). Lastly, we recombined their biphones to create six other novel words that had slightly less probable, but still high, phonotactic probabilities (labeled PP−; Table 1). For a full description of the phonotactic calculations, see Dal Ben et al. (2021) and Vitevitch and Luce (2004).

Languages were synthesized using the MBROLA speech synthesizer with a Portuguese female voice^3^ (Dutoit et al., 1996). Prosodic cues were minimized by setting the pitch constant at 180 Hz, the intensity at 77 dB, and the duration of each word to 696 ms (cf. Cunillera, Laine, et al., 2010). The total duration of each language was 15 min 39 s and 424 ms.

Following our previous studies, TPs and phonotactics were combined to create three languages. The Balanced language had test words (TP = 1.0) and part-words (TP = 0.5) with balanced phonotactic probabilities (*M*_*words*_ = 0.0072, *M*_*part-words*_ = 0.0075; Table 1); this language served as a control. The Aligned language had test words with higher phonotactic probabilities in comparison to part-words (*M*_*words*_ = 0.0085, *M*_*part-words*_ = 0.0072; Table 1). Thus, both TPs and phonotactics signaled word boundaries. Finally, in the Conflict language: test words had lower phonotactic probabilities in comparison to part-words (*M*_*words*_ = 0.0072, *M*_*part-words*_ = 0.0085; Table 1). Thus, TPs highlighted word boundaries whereas phonotactics highlighted part-words.

#### Visual Stimuli.

Six novel objects, used by Dal Ben et al. (2022), were also used in the present experiment. They were realistic, colorful, 3D objects that are part of the NOUN object base (Horst & Hout, 2016) and were chosen based on their high degree of novelty (*M* = 77%) and discriminability (*M* = 90%). For each language, objects and words were randomly paired, forming six word-object pairs. All stimuli are openly available at https://osf.io/rs2bm/.

#### Design.

**Figure 1.**
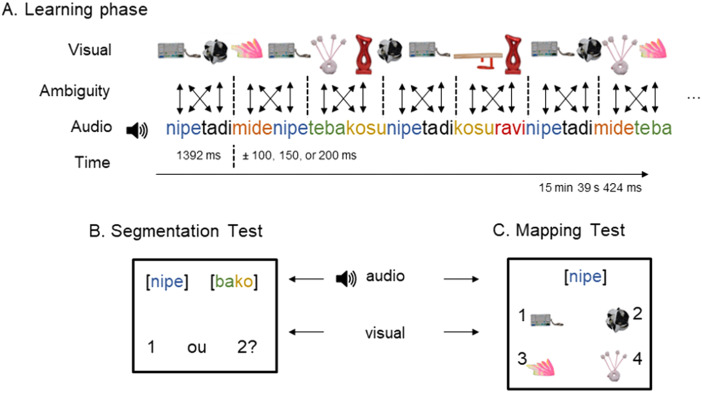
(A) Displays the Familiarization phase, with dynamic trials combining the continuous speech stream with two objects at a time. (B) Displays a trial of the speech segmentation test (two-alternative forced-choice). (C) Displays a trial of the mapping test (four-alternative forced-choice).

Our paradigm (Figure 1) was an adaptation of Cunillera, Laine, et al. (2010) and combined speech segmentation and cross-situational word learning in the same task. It had two phases: familiarization and test. During familiarization, one of the languages (Balanced, Aligned, Conflict) was played while objects were displayed on the computer screen. We matched words from the speech stream and objects on the screen in such a way that, at any given time, two objects were displayed while their corresponding words were presented (≅ 1392 ms; Figure 1). For instance, when the first word was first presented, the objects corresponding to the first and second words were displayed; when the third word was played, the first two objects were replaced by two other objects and so on. This created a highly dynamic adaptation of the classic 2 × 2 cross-situational word learning arrangement (for a video sample, see https://osf.io/rs2bm/; cf. Smith & Yu, 2008). Importantly, the onset and offset of the words and objects were desynchronized (± 100, ± 150, or ± 200 ms) to avoid additional cues to speech segmentation (Cunillera, Càmara, et al., 2010). In addition, the entire audio stream had a fade-in and fade-out effect of 500 ms to minimize cues for the initial and final words’ boundaries. Finally, to minimize fatigue from this extensive exposure (a total of 1350 word-object presentations, or 675 2 × 2 “trials”, over ≅ 15 minutes), we divided the familiarization into five blocks. Each block had 270 word-object presentations—60 for each high frequency word-object pair and 30 for each low frequency pair—and lasted a little over 3 minutes. Between blocks, participants were given a 5-second pause on a screen displaying the task progress (e.g., “Block 2 of 5”).

Following familiarization, two tests were performed, always in the same order: segmentation and mapping. The segmentation test followed a two-alternative forced-choice structure. On each trial, a frequency-balanced word (i.e., a low frequency word, TP = 1, 150 repetitions) and a part-word (TP = 0.5, 150 repetitions) were played with a pause of 500 ms between them. Participants were prompted to indicate which one was a word from the speech stream they had just heard. The order of presentation of words and part-words was counterbalanced across trials. Each of the three low frequency words were tested six times across 18 test trials, with each word being tested against each part-word twice^4^.

The mapping test followed a four-alternative forced-choice structure. Each trial began with four objects displayed in the corners of the screen: one target object (co-occurrence probability = 1 with target word) and three distractors (co-occurrence probability = 0.2 with target word). After 1 second, a target word was played and participants were prompted to select the matching object. Each of the 6 word-object pairs (3 high frequency words and 3 low frequency words) were tested twice across 12 trials.

#### Procedure.

The experiment was conducted in a sound-attenuated room and was computer administered using Psychopy2 (Peirce et al., 2019). Auditory stimuli were played on high-definition neutral headphones (AKG K240 powered by Fiio e10K dac/amp). All responses were entered on an adapted numeric keyboard with only the keys: 1, 2, 3, 4, Return, +, and − (to increase or decrease the audio volume). At the beginning of the experiment, music with the same intensity as the experimental stimuli (77 dB) was played and participants were instructed to adjust the volume to a comfortable level.

Next, they were instructed that they would hear a new language and see new objects and that their task was to discover which words corresponded to which objects. Following familiarization, they were tested on segmentation and mapping. The first two trials of each testing phase were warm-up trials used to familiarize participants with the structure of the tasks. For example, before the segmentation test trials began, participants were presented with two practice trials with a common word from Brazilian-Portuguese versus a nonsense word (e.g., pato [duck] vs. tafi). Similarly, before the mapping test trials began, participants were presented with two practice trials during which they heard a familiar word and were presented with 4 familiar objects (e.g., “pato” + picture of a duck, house, cat, ball). In addition, after each test phase, participants were asked to estimate their performance by indicating if the percentage of correct responses was between 0–25%, 25–50%, 50–75%, or 75–100%. Participants’ compliance to instructions was continuously assessed using a CCTV system. At the end, participants answered a questionnaire about their educational background and language abilities.

#### Data Analysis.

After excluding inattentive responses, defined as test trials with reaction times greater than 3 *SD*s away from the mean (segmentation: 15 trials, 1% of the data; mapping: 17 trials, 2% of the data), we fitted mixed-effects logistic regressions using the lme4 package for R (Bates et al., 2015; R Core Team, 2021) and Spearmans’ correlations, also in R, to explore speech segmentation performance, cross-situational word learning performance, relationships between them, and self-evaluation. Specific models, outcomes, and predictors are described in the next section. Given the exploratory nature of our investigation, we report effect size estimations and confidence intervals, but not *p*-values (Scheel et al., 2021). All scripts and data are openly available at https://osf.io/rs2bm/.

### Results and Discussion

#### Speech Segmentation.

To analyze speech segmentation performance, our mixed-effects logistic regression had selection of the target word (either correct or incorrect) as our outcome variable and chance level (logit of 0.5) and language (Balanced, Aligned, Conflict, respectively) as predictor variables. Our initial model had a maximal random structure with stimuli as random slopes and participants as random intercepts^5^ (Barr et al., 2013), but this model did not converge. We then pruned it to include only random intercepts for stimuli and participants^6^.

**Figure 2.**
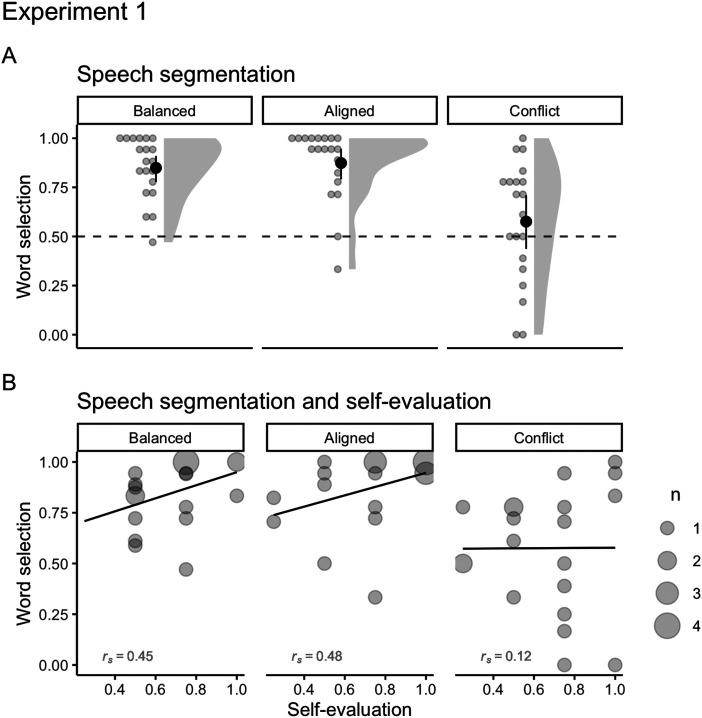
(A) Mean number of correct word selections for Balanced (*M* = 0.84, *SD* = 0.15), Aligned (*M* = 0.87, *SD* = 0.18), and Conflict (*M* = 0.57, *SD* = 0.3) languages on segmentation test of Experiment 1. Solid points represent the overall mean, error bars represent 95% CIs (non-parametric bootstrap). Points represent the mean for each participant. Shaded areas depict the distribution of individual responses. The dashed line displays the chance level (0.5). Panel B: Correlations between segmentation and self-evaluation (upper panel; *r*_*s Balanced*_ = 0.45; *r*_*s Aligned*_ = 0.48; *r*_*s Conflict*_ = 0.12) for Balanced, Aligned, and Conflict languages on Experiment 1. The size of dots indicates the number of participants that overlap in given coordinates (from 1 to 4).

Participants from the Balanced language were much more likely to select the words over the part-words at test (*Odds Ratio* = 10.95, 95% CI [4.19, 28.57]^7^; *M* = 0.85, *SD* = 0.16; Figure 2). Participants from the Aligned language, in which both TP and phonotactic probability pointed to word boundaries, were even more likely to select words over part-words (change in *OR* = 1.61, 95% CI [0.41, 6.27]; *M* = 0.87, *SD* = 0.18). On the other hand, participants from the Conflict language, in which TP and phonotactic probabilities worked against each other, were equally likely to select words and part-words (change in *OR* = 0.13, 95% CI [0.04, 0.42]; *M* = 0.57, *SD* = 0.3). These results are in line with our previous findings that adults not only track both TP and PP at the same time, but that these statistics can be combined to improve (i.e., Aligned language) or impair (i.e., Conflict language) speech segmentation (Dal Ben et al., 2021).

In addition, segmentation performance and self-evaluation (Figure 2) were positively correlated for the Balanced (*r*_*s*_ = 0.45) and Aligned (*r*_*s*_ = 0.48) languages, but not for the Conflict language (*r*_*s*_ = 0.12). This suggests that being exposed to a continuous speech in which TPs and PPs were either aligned or balanced within words formed clearer word representations, which allowed participants to estimate their knowledge of the words more accurately from the speech.

To explore whether our joint task impacts speech segmentation, we compared the present data with data from a previous investigation testing speech segmentation only (Dal Ben et al., 2021). Because we used the exact same languages as previous studies, we fit separate mixed-effects logistic regressions^8^ for each language (Balanced, Aligned, Conflict), having the selection of target words (correct or incorrect) as our outcome variable, experiment (segmentation only or simultaneous task) as a predictor variable, and participants as random intercepts.

**Figure 3.**
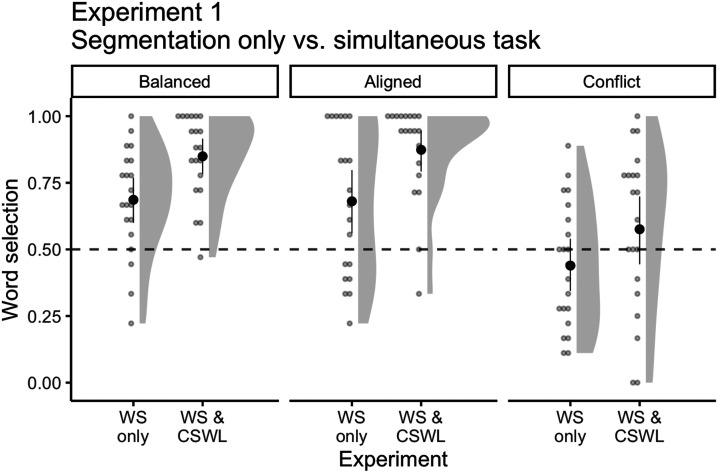
Mean number of correct word selections for Balanced (separate: *M* = 0.68, *SD* = 0.2; simultaneous: *M* = 0.84, *SD* = 0.15), Aligned (separate: *M* = 0.68, *SD* = 0.27; simultaneous: *M* = 0.87, *SD* = 0.18), and Conflict (separate: *M* = 0.43, *SD* = 0.23; simultaneous: *M* = 0.57, *SD* = 0.3) languages for an experiment testing speech segmentation only (WS only; Dal Ben et al., 2021) and on our current simultaneous task (WS & CSWL). Solid points represent the overall mean, error bars represent 95% CIs (non-parametric bootstrap). Points represent the mean for each participant. Shaded areas depict the distribution of individual responses. Dashed line displays the chance level (0.5).

For the Balanced language, participants in the simultaneous task were approximately three times more likely to choose the target word compared to the separate task (change in *OR* = 3.21, 95% CI [1.50, 6.88]; Figure 3). The difference was even higher for the Aligned language, participants from the simultaneous task were almost five times more likely to make correct selections in comparison to the separate task (change in *OR* = 4.93, 95% CI [1.34, 18.16]). On the other hand, in the Conflict language, although participants in the simultaneous task still outperformed participants from the separate task, the improvement was much less pronounced (change in *OR* = 1.96, 95% CI [0.83, 4.64]).

These results show that adults will use any statistic available–phonetic and audiovisual co-occurrences–to find words in continuous speech. Moreover, the improvement in segmentation in our current task indicates that adults benefit from tracking multiple statistical sources. This provides initial empirical support for the model proposed by Räsänen and Rasilo (2015) and is in line with recent research on language development in natural environments (Clerkin et al., 2017; Smith et al., 2018; Yu et al., 2021).

#### Cross-situational Word Learning.

To analyze cross-situational word learning, our mixed-effects logistic regression^9^ had selection of the target object (either correct or incorrect) as the outcome variable, chance level (logit of 0.25), language (Balanced, Aligned, Conflict, respectively), the frequency of word-object pairs (low or high), and their interaction as predictor variables, and stimuli and participants as random intercepts.

**Figure 4.**
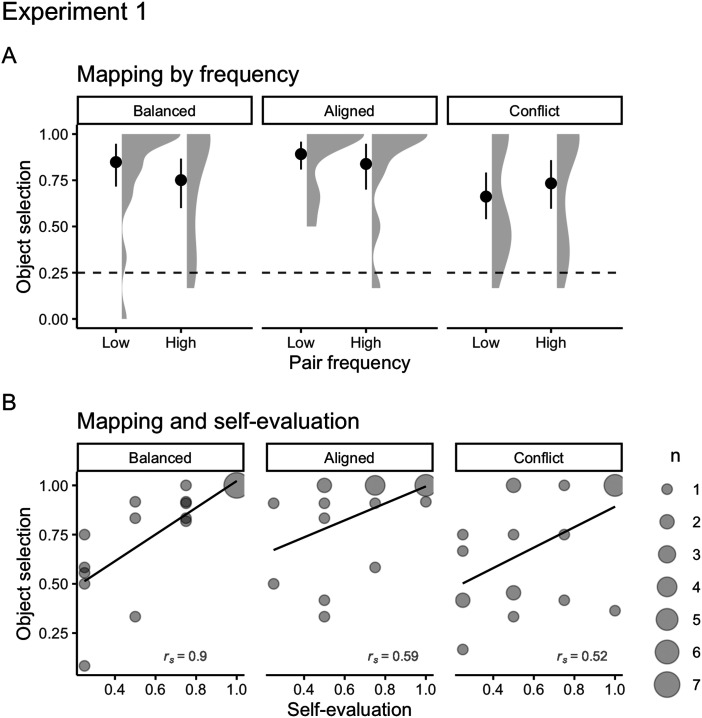
(A) Mean number of correct high and low frequency object selections for Balanced, Aligned, and Conflict languages on cross-situational word learning test of Experiment 1 (Balanced: *M*_*low*_ = 0.85, *SD* = 0.26, *M*_*high*_ = 0.75, *SD* = 0.3; Aligned: *M*_*low*_ = 0.89, *SD* = 0.18, *M*_*high*_ = 0.84, *SD* = 0.28; Conflict: *M*_*low*_ = 0.49, *SD* = 0.31, *M*_*high*_ = 0.56, *SD* = 0.32). Solid points represent the overall mean, error bars represent 95% CIs (non-parametric bootstrap). Shaded areas depict the distribution of individual responses. The dashed line displays the chance level (0.25). (B) Correlations between cross-situational word learning and self-evaluation for Balanced, Aligned, and Conflict languages (*r*_*s Balanced*_ = 0.9; *r*_*s Aligned*_ = 0.59; *r*_*s Conflict*_ = 0.52) on Experiment 1. The size of dots indicates the number of participants that overlap in given coordinates (from 1 to 7).

Across all languages and pair frequencies, participants were much more likely to select the correct object in comparison to the distractors (Figure 4; full regression table available at https://osf.io/rs2bm/). Mapping and self-evaluation (Figure 4) were positively correlated for all languages. They were strongly correlated for the Balanced language (*r*_*s*_ = 0.9), and moderately for the Aligned (*r*_*s*_ = 0.59) and the Conflict languages (*r*_*s*_ = 0.53). This suggests that participants from all languages were able to form clear word-object relationships. It was surprising to see that participants from the Conflict language, who performed at chance on the speech segmentation task, were able to form strong word-object relationships—a point to which we return later.

To explore whether our simultaneous task impacts mapping performance, we compared the present data with data from a previous experiment that only tested cross-situational word learning but using the same stimuli and population (Dal Ben et al., 2022). We fitted one mixed-effect logistic model that had mapping (correct or incorrect) as the outcome variable, the interaction between experiment (separate or simultaneous task) and language (Balanced, Aligned, Conflict) as a predictor, and participants as random intercepts^10^.

**Figure 5.**
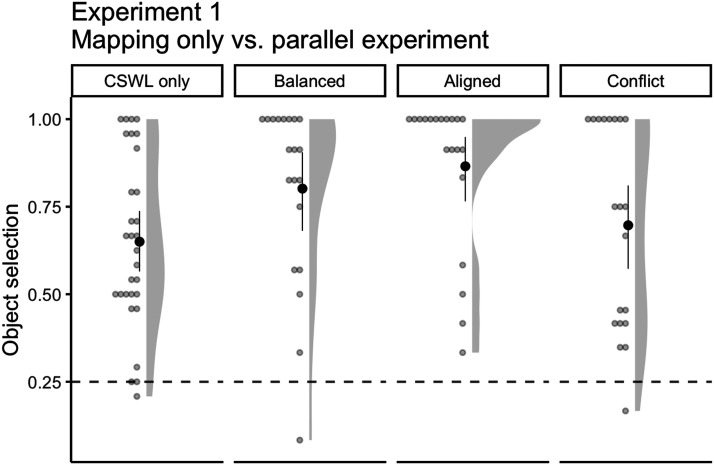
Mean number of correct object selections in an experiment testing cross-situational word learning only—CSWL only (*M* = 0.65, *SD* = 0.24; Dal Ben et al., 2022)—and in the Balanced (*M* = 0.79, *SD* = 0.28), Aligned (*M* = 0.86, *SD* = 0.23), and Conflict (*M* = 0.69, *SD* = 0.3) languages from the present, simultaneous, experiment. Solid points represent the overall mean, error bars represent 95% CIs (non-parametric bootstrap). Points represent the mean for each participant. Shaded areas depict the distribution of individual responses. Dashed line displays the chance level (0.25).

Overall, cross-situational word learning improved for all languages during the parallel task in comparison to the separate task (Figure 5). The improvement was greater for participants from the Aligned language (change in *OR* = 7.39, 95% CI [2.10, 25.98]), followed by participants from the Balanced language (change in *OR* = 3.34, 95% CI [1.37, 5.52]). Although less pronounced, there was also an improvement for the Conflict language (change in *OR* = 1.60, 95% CI [0.50, 5.05]), which indicates that participants can benefit from word-object co-occurrence even when TP and phonotactics point to different word boundaries.

#### Relationship Between Speech Segmentation and Word Mapping.

**Figure 6.**
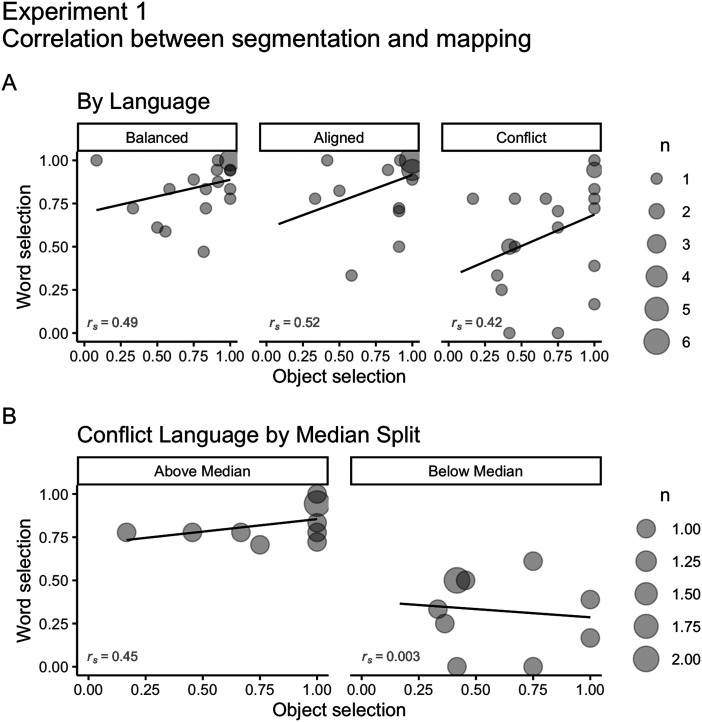
(A) Correlations between speech segmentation and mapping for Balanced, Aligned, and Conflict Languages on Experiment 1 (*r*_*s Balanced*_ = 0.49; *r*_*s Aligned*_ = 0.52; *r*_*s Conflict*_ = 0.42). The size of dots indicates the number of participants that overlap in each coordinate (from 1 to 6). (B) Correlations between speech segmentation and mapping in the Conflict Language for participants with speech segmentation above the median (*Mdn* = 0.66, *IQR* = 0.4; *r*_*s*_ = 0.45) and below the median (*r*_*s*_ = 0.003). The size of dots indicates the number of participants that overlap in each coordinate (from 1 to 2).

To explore potential relationships between speech segmentation and word mapping, we ran Spearmans’ correlations between words’ and objects’ selections (average scores per participant) for each Language. We found moderate positive correlations between segmentation and mapping for all Languages (*r*_*s Balanced*_ = 0.49; *r*_*s Aligned*_ = 0.52; *r*_*s Conflict*_ = 0.42; Figure 6A). Overall, participants that were better at segmentation were also better at mapping. To further explore if that was true for participants from the Conflict Language, we performed a median split of segmentation performance (*Mdn* = 0.66, *IQR* = 0.4) and ran Spearman correlation tests for each group separately (Figure 6B). Participants that successfully segmented the speech (above median) were also successful in mapping words to objects (*r*_*s*_ = 0.46). However, we found no relationship between segmentation and mapping for those who performed poorly on segmentation (below the median; *r*_*s*_ = 0.003).

Our design does not inform us about potential learning sequences. Intuitively, strong speech segmentation skills should lead to strong word mapping, which is confirmed to some extent by the positive correlation between word and object selections for participants above the median in the Conflict language, but not for those below the median. Interestingly, simulations by Räsänen and Rasilo (2015) favor a simultaneous performance in which speech segmentation and mapping retrofeed each other, driving performance on both tasks. In this regard, the absence of a relationship between segmentation and mapping for participants below the median in the Conflict Language indicates that these performances could be independent from one another.

Overall, results from the present experiment suggest that not only can adults simultaneously track conditional probabilities between audio and visual stimuli to segment words from continuous speech streams and map them to referents under ambiguous learning contexts, but that both segmentation and mapping improve when a greater set of cues, even from different modalities, are available (Figures 3 and 5). Such results provide preliminary empirical evidence to the model of simultaneous segmentation and ambiguous mapping proposed by Räsänen and Rasilo (2015) and Jones et al. (2010).

Our results also indicate that phonotactic probabilities, or how familiar syllables’ positional probabilities are in the native language of the participants, also impact such joint performance. When transitional and phonotactic probabilities worked together to signal word boundaries, segmentation and mapping improved (Aligned language) in contrast to when the phonotactic probabilities were balanced among test items (Balanced language). However, the impact of phonotactics was most pronounced when it conflicted with TP information. In the Conflict language, overall, participants failed to show a preference for words when compared to part-words at test (Figure 2). Nonetheless, they were able to map words and objects (Figure 4). How could this happen?

If we assume that segmentation is a necessary pre-step to cross-situational mapping, then this result is hard to explain. However, if adults use whatever informative statistics they have at hand to solve linguistic ambiguity, they would take advantage of both transitional and phonotactic statistics and word-object co-occurrences in the Aligned and Balanced languages. On the other hand, in the Conflict language, statistics were not consistent enough to promote segmentation, but co-occurrences between word syllables and objects were consistent enough to promote mapping and, to some extent, speech segmentation—even without clear and explicit word representations. It is worth noting that objects were consistently paired with words only, and not with part-words. This might have provided some participants with enough information for speech segmentation. It might also have decreased the influence of statistical cues on segmentation (both TPs and PPs). Nonetheless, if word-object co-occurrence was the main source of information for speech segmentation, we should have seen similar levels of segmentation in all languages.

Moreover, our two-alternative forced-choice test might not have been sensitive enough to capture the weaker and implicit word representations that might have arisen in the Conflict language, providing us a partial picture of participants’ speech segmentation. Our two-alternative-forced-choice trials contrasted words with stronger TPs and weaker phonotactic probability, or part-words with weaker TPs but stronger phonotactic probability. The contrast between recently acquired TP knowledge, and language specific phonotactic knowledge learned across the lifespan, may have impaired word selection (Finn & Hudson Kam, 2008). With this in mind, we replicate the current experiment, but using an arguably more sensitive speech segmentation measurement.

Finally, it is worth noting that our careful selection and combination of syllables to create disyllabic words with varying TP and PP contrasts introduced an important confound to our study: none of our words shared syllables. As all syllables were unique to a given word, tracking co-occurrences between individual syllables and objects would be enough to solve the mapping task—but not the segmentation task. This learning strategy would greatly reduce mapping complexity: participants could ignore half of the syllables and all linguistic regularities (i.e., TP and PP). Whereas this strategy may be computationally most simplistic, it seems like, as a group, participants in the Balanced and Aligned languages did track word-level statistics, as indicated by their segmentation performance. However, when faced with conflicting linguistic regularities, participants in the Conflict language might have defaulted to this more simplistic learning strategy and solved the mapping task without relying on word representations. Importantly, this confound extends to Experiment 2 and we further discuss it in the General Discussion.

## EXPERIMENT 2

In an attempt to capture the potentially nuanced word form knowledge implicitly arising from experience with the Conflict language, in the current experiment we use a more sensitive word segmentation test: go/no-go (François et al., 2017). In this test, each item is presented and evaluated separately, one at a time. By avoiding the contrast between stimuli (i.e., word, part-word, non-word) with different statistics (TP and phonotactics) at test and by adding a new stimuli type (i.e., non-words), we aim for a more fine-grained understanding of word representations in the Conflict language. The mapping test is the same as in Experiment 1.

### Method

This experiment was a replication of Experiment 1, but it was fully online due to the COVID-19 pandemic. Differences in methodology are described below.

#### Participants.

Forty-five adults, all native speakers of Brazilian-Portuguese, with no reported visual or auditory impairment that could interfere with the task, participated. However, 10 participants were excluded from the final analyses because they failed or missed attention check questions, reported using their mobile phones or taking notes during the experiment (see Data analysis for further details). The final sample consisted of 35 adults (*M*_*age*_ = 23.51, ± 4.01 *SD*, 22 female). As in Experiment 1, participants were recruited at the official Facebook group of the Universidade Federal de São Carlos and received no compensation for their participation. The study was conducted according to the Declaration of Helsinki and the Ethics Committee of the host university approved the research (#3.085.914).

#### Stimuli and Design.

We used the Conflict language from Experiment 1, with the same word-object pairs. As a brief reminder, in this Language, words had high TPs (TP = 1; Table 1) and lower phonotactic probabilities (*M*_*words*_ = 0.0072), while part-words had lower TPs (TP = 0.5) and higher phonotactic probabilities (*M*_*part-words*_ = 0.0085). In addition, we created three additional non-words with balanced phonotactic by recombining the initial syllables of words (i.e., /visu/, /tami/, /rako/; PPs = 0.0080, 0.0074, 0.0069, respectively). Because their syllables never occurred together in the Language, their TP was zero.

A similar design from Experiment 1 was used here, with four main differences. First, given the online nature of the study, before beginning the experimental task, participants were instructed to move to a quiet room, to turn off any electronic devices (e.g., cellphone, TV), to wear earphones, and not to take notes during the experiment. Second, the segmentation test followed a go/no-go structure: test words (i.e., /nipe/, /tadi/, /mide/), part-words (i.e., /sute/, /viko/, /bara/), and non-words (i.e., /visu/, /tami/, /rako/) were presented one at a time and participants were instructed to indicate whether they were or were not words from the language they had just heard (by pressing to “s” or “n”, corresponding to “sim” [yes] or “não” [no] in Portuguese). Each stimuli was tested 6 times (total of 54 trials). Third, attention checks were conducted during the familiarization and segmentation test. At each familiarization block, participants were prompted to answer five simple questions (i.e., “Are you alive?”, “Are you sleeping?”, “Are you breathing?”, “Are you dead?”, “Are you awake?”). Between segmentation test trials, attention checks displayed either a Portuguese word or a made-up word (e.g., “mesa” [table], “drevo”) printed on the screen and participants were prompted to indicate if the word existed in Portuguese or not. During both familiarization and test, participants indicated their answers for attention checks by pressing the “s” or “n” keys on the keyboard. Fourth, at the end of the experiment, we checked for compliance to instructions by asking participants whether they had used the cellphone or if they had taken notes during the experiment.

#### Procedure.

The experiment was entirely online, hosted on Pavlovia and programmed using Psychopy3 (Bridges et al., 2020). After agreeing to participate, participants were instructed to avoid distractions (see previous section), answered a questionnaire about their educational background and language abilities, and then started the experimental task. As in Experiment 1, they were exposed to three phases: familiarization, segmentation test, and mapping test (same as Experiment 1). In addition, attention checks (described before) were presented between familiarization blocks and between trials during the segmentation test.

#### Data Analysis.

We followed similar analytical steps from Experiment 1. We first excluded participants who reported using their mobile phones during the experiment (*n* = 3) and those (*n* = 2) who failed two or more attention checks (out of five questions) during familiarization. Another five participants were excluded because their reaction times to attention checks in the familiarization or segmentation tests were greater than 3 *SD*s from the mean. For the remaining participants (*n* = 35), we excluded trials with reaction times greater than 3 *SD*s away from the mean (segmentation: 32 trials overall, 1% of the data; mapping: 7 trials overall, 1% of the data). The final data was entered in mixed-effect logistic regressions. The outcome, predictors, and random effects for each model is described in the next section.

### Results and Discussion

#### Speech Segmentation.

To analyze speech segmentation, we fitted a mixed-effects logistic regression with words’, part-words’, and non-words’ evaluations as the outcome variable. Selection of words and rejections of part-words and non-words were coded as correct responses. Predictors were the chance level (logit of 0.5) and stimuli type (words, part-words, non-words), stimuli and participants were random intercepts^11^.

**Figure 7.**
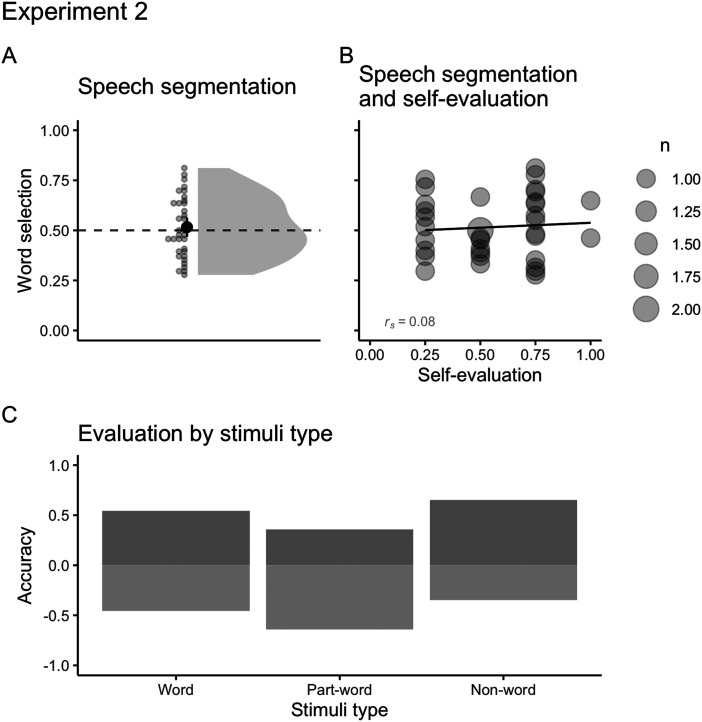
(A) Mean number of correct word selections and part-word and non-word rejections on Experiment 2 (*M* = 0.51, *SD* = 0.15). The solid point represents the overall mean, error bars represent 95% CIs (non-parametric bootstrap). Points represent the mean for each participant. The shaded area depicts the distribution of individual responses. The dashed line displays the chance level (0.5). (B) Correlations between segmentation and self-evaluation (*r*_*s Conflict*_ = 0.08) on Experiment 2. The size of dots indicates the number of participants that overlap in given coordinates (from 1 to 2). (C) Evaluation by stimuli type (word, part-word, non-word). Positive scores represent correct selection of words (*M* = 0.54, *SD* = 0.28) and rejection of part-words (*M* = 0.35, *SD* = 0.25) and non-words (*M* = 0.65, *SD* = 0.27). Negative scores represent incorrect rejections of words and selection of part-words and non-words.

We replicated the results from Experiment 1 – Conflict language. Overall, participants’ performance was at chance level (*M* = 0.51, *SD* = 0.15; Figure 7A). The analyses by stimuli type (Figure 7C) reveal a slight tendency for evaluating words as such (*OR* = 1.21, 95% CI [0.61, 2.4]), a stronger tendency for correctly rejecting non-words (change in *OR* = 1.68, 95% CI [0.67, 4.2]), and a much less accurate judgment when rejecting part-words (change in *OR* = 0.41, 95% CI [0.16, 1.03]). As in Experiment 1, there was no correlation between speech segmentation and self-evaluation (*r*_*s Conflict*_ = 0.08).

These results indicate that participants might have tracked both transitional and phonotactic statistics from familiarization, but used them differently when evaluating stimuli during test. For instance, they might have relied on TP information when evaluating words (higher TP and lower phonotactics) and phonotactic information when evaluating part-words (lower TP and higher phonotactics). Finally, the lack of familiarity with non-words (no TP information), and the balanced phonotactic statistics, might have generated correct non-word rejections. Overall, our nuanced results could indicate that the go/no-go procedure is not sensitive enough to capture implicit word representation arising from speech segmentation of a language with conflicting statistics—a point we return to in the General Discussion.

#### Cross-situational Word Learning.

**Figure 8.**
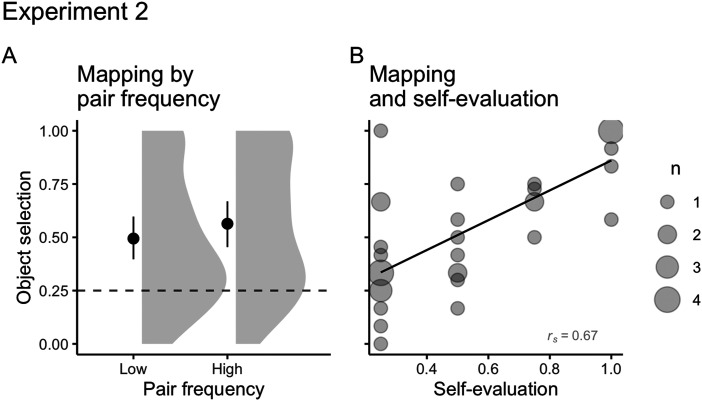
(A) Mean number of correct object selections for high (*M* = 0.56, *SD* = 0.31) and low (*M* = 0.49, *SD* = 0.31) frequency pairs on Experiment 2. The solid point represents the overall mean, error bars represent 95% CIs (non-parametric bootstrap). The shaded area depicts the distribution of individual responses. The dashed line displays the chance level (0.25). (B) Correlations between mapping and self-evaluation (*r*_*s Conflict*_ = 0.67) on Experiment 2. The size of dots indicates the number of participants that overlap in given coordinates (from 1 to 4).

To model mapping performance, our mixed-effect logistic regression had object selection (correct or incorrect) as the outcome variable, chance level (logit of 0.25) and target stimuli frequency (150 or 300 repetitions) as predictors, and stimuli and participants as random intercepts^12^. As in Experiment 1, participants correctly mapped both high and low frequency words above chance level (*M*_*high*_ = 0.56, *SD*_*high*_ = 0.31; *M*_*low*_ = 0.49, *SD*_*low*_ = 0.31; Figure 8), with small differences in the likelihood of correctly selecting high or low frequency word-object pairs (*OR*_*high*_ = 1.51, 95% CI [0.75, 3.02]; change in *OR*_*low*_ = 0.68, 95% CI [0.35, 1.36]). Again, we found a moderate positive correlation between mapping and self-evaluation (*r*_*s*_ = 0.67; Figure 8).

#### Relationship Between Speech Segmentation and Word Mapping.

**Figure 9.**
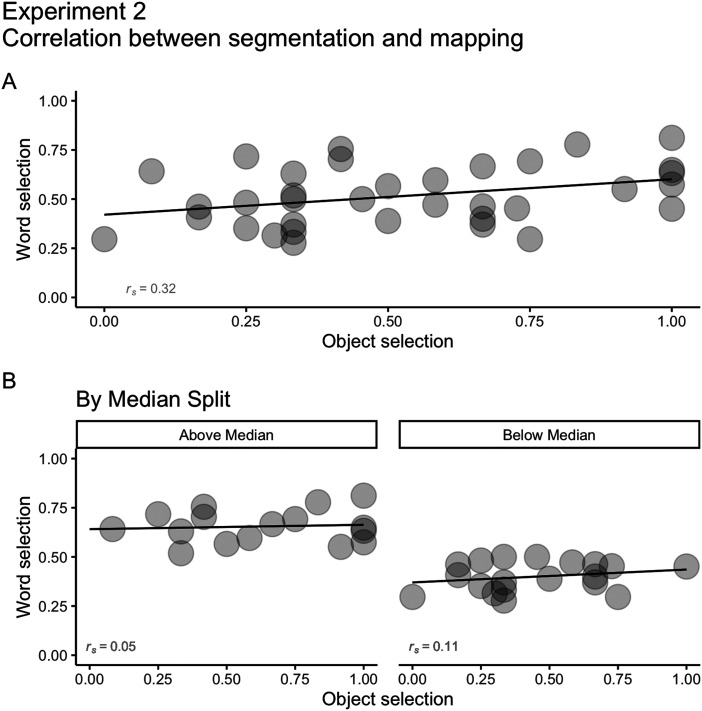
(A) Correlation between segmentation and mapping (*r*_*s*_ = 0.32) on Experiment 2. (B) Correlations between speech segmentation and mapping for participants with speech segmentation above the median (*Mdn* = 0.5, *IQR* = 0.24; *r*_*s*_ = 0.05) and below the median (*r*_*s*_ = 0.11).

As in Experiment 1, we ran Spearmans’ correlation tests between words’ and objects’ selections (average scores per participant) to explore potential relationships between speech segmentation and word mapping. We found a weak positive correlation between segmentation and mapping (*r*_*s*_ = 0.32; Figure 9). Again, overall, participants that were better at segmentation were also better at mapping. Further exploration by speech segmentation median split (*Mdn* = 0.5, *IQR* = 0.24) revealed little difference between participants above the median (*r*_*s*_ = 0.05) and below the median (*r*_*s*_ = 0.11), with no correlation between segmentation and mapping for both groups.

The current experiment was designed to further evaluate the effects of the conflict between transitional and phonotactic statistics on simultaneous speech segmentation and cross-situational word learning. Overall, we replicated Experiment 1: speech segmentation, as measured by a go/no-go test, was at chance level, but word-object mapping performance was above chance. Nonetheless, our more sensitive word segmentation test provided some nuanced information about stimulus representations.

We found that participants were likely to correctly evaluate non-words as such. This indicates that how participants represented words and part-words was most likely the result of the interplay between phonotactic and transitional probabilities. For instance, stronger phonotactics combined with a probabilistic transitional probability (TP = 0.5) lead participants to incorrectly evaluate part-words as words. On the other hand, the weaker phonotactics combined with deterministic transitional information (TP = 1) prompt only a slight tendency to correctly evaluate words as such.

As in Experiment 1, speech segmentation performance and self-evaluation indicate that the conflict between transitional and phonotactic probabilities impaired the formation of clear word representations, which could have impaired participants’ accuracy when estimating their knowledge of words from speech. Again, however, despite the absence of clear word representations, participants were able to map words to objects. Consistent word-object co-occurrences might have provided sufficient information to promote mapping and some level of segmentation, despite conflicting phonetic information (Räsänen & Rasilo, 2015). Furthermore, as in Experiment 1, syllables were not shared between words. Participants could have tracked co-occurrences between individual syllables and objects to solve the mapping task, without relying on any word-level phonetic information. Whereas using this strategy would allow participants to solve the mapping task, it wouldn’t allow them to gain any word-level information. Thus, if this was the entirety of the explanation, speech segmentation performance should have been at chance level for words, part-words, and non-words. However, during the go/no-go test, participants were more likely to correctly select words as such and to correctly reject non-words, indicating that they tracked word-level statistics to some degree.

Next, we discuss a possible design to overcome this important confound as well as some of the limitations of our study. Moreover, we, discuss how our preliminary findings broaden our understanding of statistical learning from multiple cues and prompt further research on the subject.

## GENERAL DISCUSSION

In the present study, we explored whether adults could segment speech streams into words and map them to objects simultaneously by tracking conditional probabilities across ambiguous presentations. We also investigated the effects of word-level phonotactics in segmentation and mapping. Phonotactics were either balanced, aligned, or in conflict with transitional probabilities. We found that participants were successful at both the segmentation and mapping tasks when transitional and phonotactic probabilities were either aligned or balanced across words. In contrast, when transitional and phonotactic probabilities were in conflict, we did not find evidence for speech segmentation, but we still found evidence for mapping.

Our results offer preliminary support for the idea that a greater set of cues, even in different modalities, supports speech segmentation and word learning in ambiguous situations (Jones et al., 2010; Räsänen & Rasilo, 2015). Not only were participants able to segment and map words simultaneously by tracking conditional probabilities, but their overall performance was stronger in this simultaneous task in comparison to separate tasks of segmentation and cross-situational word learning. This adds to the evidence showing that language learners benefit from combining several sources of linguistic information when learning a new language (Choi et al., 2018; Johnson, 2016; Saffran, 2020; Smith et al., 2018; Tachakourt, 2023; Yurovsky et al., 2012). This combination might be especially useful when dealing with ambiguity, as even a partial solution to one linguistic challenge could reduce ambiguity in other linguistic challenges (for related evidence, see Feldman, Griffiths, et al., 2013; Feldman, Myers, et al., 2013).

Our design does not provide insights into specific learning strategies used by our participants. For instance, they could have used an aggregation strategy, gradually segmenting speech and using the segmented words as anchors for further segmentation and mapping. Or they could have used hypothesis testing from the start, electing syllable sequences and testing their co-occurrence with each other and with objects over time. Participants might have also used a blend of these strategies depending on the level of ambiguity they were facing (Yurovsky & Frank, 2015). In addition, our selection of unique syllables for each word introduced an important confound to our study. Participants could have solved the mapping task even when ignoring half of the syllables and all word-level statistics. In contrast, more efficient segmentation performance for all languages (except for the Conflict language on Experiment 1) suggests that participants tracked word-level statistics to some degree. Furthermore, the positive correlation between segmentation and mapping, found in all languages of both experiments, suggests that both speech segmentation, that relied on word-level statistics, and cross-situational mapping were in close interaction, potentially retro feeding each other over time (in line with proposals by Räsänen & Rasilo, 2015).

Interestingly, the conflict between phonotactics and transitional information might have impaired the formation of clear and explicit word representations, but not the formation of strong word-object relationships. Whereas this could point to independent processing of phonetic and audiovisual statistics, it could also be that participants did form clear, but implicit, word representations that our explicit measurements (either a two-alternative forced-choice or go/no-go) were not able to capture. For instance, despite the conflict between transitional and phonotactic statistics, participants were still able to consistently reject non-words during Experiment 2, showing that stimuli with different degrees of statistical information were treated differently. It is worth reinforcing that we manipulated TPs and PPs differently. Whereas TPs were determined when designing the experimental stimuli, PPs were estimated from participants’ native language. This led to differences in strength between cues that might have had different effects on participants’ processing. For instance, they could have relied more on TP to find word boundaries and on PPs to form strong word representations. A more direct way to assess these implicit processes and to overcome the confound of not repeating syllables across words may be to use EEG measures during the passive familiarization phase.

Recording neural activity during familiarization could inform us about how the alignment or conflict between TP and PP are processed and what happens when these statistics are violated (e.g., Elmer et al., 2021; François et al., 2017). Furthermore, the use of neural entrainment analysis could provide direct evidence to whether participants track word-level statistics (disyllabic words) or individual syllables (e.g., Batterink & Paller, 2017; Choi et al., 2020). In an ongoing EEG study in one of our labs, we are measuring: a) whether participants will show similar ERPs to violations of transitional and phonotactic information presented in the Balanced and in the Conflict languages and b) the temporal entrainment of their neural activity during familiarization.

Another invaluable source of information on the learning mechanisms involved in the simultaneous speech segmentation and mapping are the cognitive processes underlying such performances. For instance, auditory and visual memory have been shown to predict cross-situational word learning (Vlach & DeBrock, 2017; Vlach & Johnson, 2013). Differences in attention have also been found to impact statistical learning (Smith & Yu, 2013; Yurovsky et al., 2013). Future research could measure these and other cognitive processes to better understand their role in statistical language learning.

Our study was exploratory in nature. Building on our promising initial findings, future replications should put our findings to the test. They could also address some of the shortcomings of the present investigation. Future investigations could, for example, manipulate both TPs and PPs in a similar way, leading to a finer control of their contrast. Both cues could be defined experimentally (e.g., Benitez & Saffran, 2021), or estimated directly from participants’ natural language. The latter estimation could lead to the design of natural continuous speech (in line with Hay et al., 2011) that comprises the variability in transitional and phonotactic probabilities that learners face in the wild, increasing the ecological validity of statistical learning findings (cf. Smith et al., 2014). Future research could also have participants from more heterogeneous backgrounds, for example, by recruiting participants from different ages, in different countries, with different socioeconomic status. We tested fairly homogenous samples of young college students from a single language background. The trends we found may not generalize to other populations (Simons et al., 2017). Also, it could be that the statistical learning mechanisms involved in this simultaneous task may have different roles across development (Choi et al., 2018; Danielson et al., 2017; Smith et al., 2018). Future research could investigate simultaneous statistical language learning across development to bridge the gaps between young adults, infants, and older adults. Finally, although highly dynamic, our task comprises only a small sampling of the challenges (i.e., segmentation and mapping) and statistics (i.e., conditional probabilities) available for language learners in natural environments. Future studies could improve ecological validity by, for instance, combining statistical, prosodic, and semantic information (Hay et al., 2011; Karaman & Hay, 2018), or diving into natural environments (Bogaerts et al., 2022; Yu et al., 2021).

Learning languages is difficult. To overcome many linguistic challenges, learners can rely on several cues. Here we provide preliminary evidence that adults can track conditional probabilities to simultaneously find words in continuous speech and map them to objects across ambiguous situations. We also show that the level of pre-experimental familiarity with words can impact their representation. By doing so, we contribute to a more nuanced understanding of how statistical cues interact to promote language learning.

## ACKNOWLEDGMENTS

This work was supported by grants from FAPESP (#2015/26389-7, #2018/04226-7) and CAPES (#001) to RDB; FAPESP (#2018/18748-5) to ITP; from INCT-ECCE (National Institute on Cognition, Behavior and Teaching; CNPq #573972/2008-7, #465686/2014-1, FAPESP #2008/57705-8, #2014/50909-8) to DHS; and from the NICHD (#R01HD083312) to JFH. RDB is now at Ambrose University.

## AUTHOR CONTRIBUTIONS

RDB: Conceptualization, Methodology, Software, Investigation, Visualization, Data Formal Analysis, Writing - Original Draft; ITP: Investigation, Data Formal Analysis, Writing - Original Draft; DHS & JFH: Conceptualization, Writing - Review & Editing, Supervision, Project administration, Funding acquisition.

## DATA AVAILABILITY STATEMENT

The materials, code, and data from this study are openly available on Open Science Framework at https://osf.io/rs2bm/.

## Notes

^1^ Phonotactic probabilities calculated using Vitevitch and Luce (2004) online calculator.^2^ Here we do not join the productive debate between hypothesis-testing and aggregation as learning mechanisms for cross-situational word learning (e.g., Yurovsky & Frank, 2015), as we believe it is beyond the scope of our study.^3^ We used the MBROLA database br4 (available at: https://github.com/numediart/MBROLA-voices).^4^ The decision to test each word six times was based on our previous investigation of speech segmentation only (Dal Ben et al., 2021). Whereas this number of repetitions is higher in comparison to similar studies (e.g., Cunillera, Laine, et al., 2010; François et al., 2017), follow-up analyses revealed that trial number did not predict performance on neither Experiment 1 nor 2. Full analysis available at: https://osf.io/rs2bm/.^5^ lme4 syntax: selection ∼ chance level + language + (stimuli|participant).^6^ lme4 syntax: selection ∼ chance level + language + (1|stimuli) + (1|participant).^7^ Regression tables are available at https://osf.io/rs2bm/.^8^ lm4 syntax for each language: word selection ∼ experiment + (1|participant).^9^ lm4 syntax: object selection ∼ chance level + language * pair frequency + (1|stimuli) + (1|participant).^10^ lme4 syntax: object selection ∼ experiment:language + (1|participant).^11^ lme4 syntax: selection ∼ chance level + stimuli type + (1|stimuli) + (1|participant).^12^ lme4 syntax: selection ∼ chance level + target frequency + (1|stimuli) + (1|participant).

## References

[bib1] Aslin, R. N., Saffran, J. R., & Newport, E. L. (1998). Computation of conditional probability statistics by 8-month-old infants. Psychological Science, 9(4), 321–324. 10.1111/1467-9280.00063

[bib2] Barr, D. J., Levy, R., Scheepers, C., & Tily, H. J. (2013). Random effects structure for confirmatory hypothesis testing: Keep it maximal. Journal of Memory and Language, 68(3), 255–278. 10.1016/j.jml.2012.11.001, 24403724 PMC3881361

[bib3] Bates, D., Mächler, M., Bolker, B., & Walker, S. (2015). Fitting linear mixed-effects models using lme4. Journal of Statistical Software, 67(1), 1–48. 10.18637/jss.v067.i01

[bib4] Batterink, L. J., & Paller, K. A. (2017). Online neural monitoring of statistical learning. Cortex, 90, 31–45. 10.1016/j.cortex.2017.02.004, 28324696 PMC5438777

[bib5] Benitez, V. L., & Saffran, J. R. (2021). Two for the price of one: Concurrent learning of words and phonotactic regularities from continuous speech. PLoS ONE, 16(6), Article e0253039. 10.1371/journal.pone.0253039, 34115799 PMC8195377

[bib6] Black, A., & Bergmann, C. (2017). Quantifying infants’ statistical word segmentation: A meta-analysis. In G. Gunzelmann, A. Howes, T. Tenbrink, & E. J. Davelaar (Eds.), Proceedings of the 39th Annual Conference of the Cognitive Science Society (pp. 124–129). Cognitive Science Society.

[bib7] Bogaerts, L., Siegelman, N., Christiansen, M. H., & Frost, R. (2022). Is there such a thing as a ‘good statistical learner’? Trends in Cognitive Sciences, 26(1), 25–37. 10.1016/j.tics.2021.10.012, 34810076

[bib8] Brent, M. R., & Siskind, J. M. (2001). The role of exposure to isolated words in early vocabulary development. Cognition, 81(2), B33–B44. 10.1016/S0010-0277(01)00122-6, 11376642

[bib9] Bridges, D., Pitiot, A., MacAskill, M. R., & Peirce, J. W. (2020). The timing mega-study: Comparing a range of experiment generators, both lab-based and online. PeerJ, 8, Article e9414. 10.7717/peerj.9414, 33005482 PMC7512138

[bib10] Cannistraci, R. A., Dal Ben, R., Karaman, F., Esfahani, S. P., & Hay, J. F. (2019). Statistical learning approaches to studying language development. In J. S. Horst & J. von Koss Torkildsen (Eds.), International handbook of language acquisition (pp. 51–75). Routledge. 10.4324/9781315110622-4

[bib11] Choi, D., Batterink, L. J., Black, A. K., Paller, K. A., & Werker, J. F. (2020). Preverbal infants discover statistical word patterns at similar rates as adults: Evidence from neural entrainment. Psychological Science, 31(9), 1161–1173. 10.1177/0956797620933237, 32865487

[bib12] Choi, D., Black, A. K., & Werker, J. F. (2018). Cascading and multisensory influences on speech perception development. Mind, Brain, and Education, 12(4), 212–223. 10.1111/mbe.12162

[bib13] Clerkin, E. M., Hart, E., Rehg, J. M., Yu, C., & Smith, L. B. (2017). Real-world visual statistics and infants’ first-learned object names. Philosophical Transactions of the Royal Society of London, Series B: Biological Sciences, 372(1711), Article 20160055. 10.1098/rstb.2016.0055, 27872373 PMC5124080

[bib14] Cox, C. M. M., Keren-Portnoy, T., Roepstorff, A., & Fusaroli, R. (2022). A Bayesian meta-analysis of infants’ ability to perceive audio–visual congruence for speech. Infancy, 27(1), 67–96. 10.1111/infa.12436, 34542230

[bib15] Cristia, A. (2018). Can infants learn phonology in the lab? A meta-analytic answer. Cognition, 170, 312–327. 10.1016/j.cognition.2017.09.016, 29102857

[bib16] Cunillera, T., Càmara, E., Laine, M., & Rodríguez-Fornells, A. (2010). Speech segmentation is facilitated by visual cues. Quarterly Journal of Experimental Psychology, 63(2), 260–274. 10.1080/17470210902888809, 19526435

[bib17] Cunillera, T., & Guilera, G. (2018). Twenty years of *statistical learning*: From language, back to machine learning. Scientometrics, 117(1), 1–8. 10.1007/s11192-018-2856-x

[bib18] Cunillera, T., Laine, M., Càmara, E., & Rodríguez-Fornells, A. (2010). Bridging the gap between speech segmentation and word-to-world mappings: Evidence from an audiovisual statistical learning task. Journal of Memory and Language, 63(3), 295–305. 10.1016/j.jml.2010.05.003

[bib19] Dal Ben, R., Souza, D. d. H, & Hay, J. F. (2019). Cross-situational word learning: Systematic review and meta-analysis. Manuscript in preparation. 10.17605/OSF.IO/GU9RB

[bib20] Dal Ben, R., Souza, D. d. H, & Hay, J. F. (2021). When statistics collide: The use of transitional and phonotactic probability cues to word boundaries. Memory & Cognition, 49(7), 1300–1310. 10.3758/s13421-021-01163-4, 33751490 PMC9651140

[bib21] Dal Ben, R., Souza, D. d. H, & Hay, J. F. (2022). Combining statistics: The role of phonotactics on cross-situational word learning. Psicologia: Reflexao e Critica, 35(1), Article 30. 10.1186/s41155-022-00234-y, 36169750 PMC9519815

[bib22] Danielson, D. K., Bruderer, A. G., Kandhadai, P., Vatikiotis-Bateson, E., & Werker, J. F. (2017). The organization and reorganization of audiovisual speech perception in the first year of life. Cognitive Development, 42, 37–48. 10.1016/j.cogdev.2017.02.004, 28970650 PMC5621752

[bib23] Dutoit, T., Pagel, V., Pierret, N., Bataille, F., & van der Vrecken, O. (1996). The MBROLA project: Towards a set of high quality speech synthesizers free of use for non commercial purposes. In Proceedings of Fourth International Conference on Spoken Language Processing (Vol. 3, pp. 1393–1396). IEEE. 10.1109/ICSLP.1996.607874

[bib24] Elmer, S., Valizadeh, S. A., Cunillera, T., & Rodriguez-Fornells, A. (2021). Statistical learning and prosodic bootstrapping differentially affect neural synchronization during speech segmentation. NeuroImage, 235, Article 118051. 10.1016/j.neuroimage.2021.118051, 33848624

[bib25] Estivalet, G. L., & Meunier, F. (2015). The Brazilian Portuguese Lexicon: An instrument for psycholinguistic research. PLoS ONE, 10(12), Article e0144016. 10.1371/journal.pone.0144016, 26630138 PMC4668042

[bib26] Feldman, N. H., Griffiths, T. L., Goldwater, S., & Morgan, J. L. (2013). A role for the developing lexicon in phonetic category acquisition. Psychological Review, 120(4), 751–778. 10.1037/a0034245, 24219848 PMC3873724

[bib27] Feldman, N. H., Myers, E. B., White, K. S., Griffiths, T. L., & Morgan, J. L. (2013). Word-level information influences phonetic learning in adults and infants. Cognition, 127(3), 427–438. 10.1016/j.cognition.2013.02.007, 23562941 PMC3646897

[bib28] Finn, A. S., & Hudson Kam, C. L. (2008). The curse of knowledge: First language knowledge impairs adult learners’ use of novel statistics for word segmentation. Cognition, 108(2), 477–499. 10.1016/j.cognition.2008.04.002, 18533142

[bib29] François, C., Cunillera, T., Garcia, E., Laine, M., & Rodriguez-Fornells, A. (2017). Neurophysiological evidence for the interplay of speech segmentation and word-referent mapping during novel word learning. Neuropsychologia, 98, 56–67. 10.1016/j.neuropsychologia.2016.10.006, 27732869

[bib30] Frank, M. C., Mansinghka, V., Gibson, E., & Tenenbaum, J. B. (2007). Word segmentation as word learning: Integrating stress and meaning with distributional cues. In H. Caunt-Nulton, S. Kulatilake, & I. Woo (Eds.), Proceedings of the 31st Annual Boston University Conference on Language Development (pp. 218–229). Boston University.

[bib31] Graf Estes, K. (2009). From tracking statistics to learning words: Statistical learning and lexical acquisition. Linguistics and Language Compass, 3(6), 1379–1389. 10.1111/j.1749-818X.2009.00164.x

[bib32] Graf Estes, K., Edwards, J., & Saffran, J. R. (2011). Phonotactic constraints on infant word learning. Infancy, 16(2), 180–197. 10.1111/j.1532-7078.2010.00046.x, 21297877 PMC3032547

[bib33] Graf Estes, K., Evans, J. L., Alibali, M. W., & Saffran, J. R. (2007). Can infants map meaning to newly segmented words? Statistical segmentation and word learning. Psychological Science, 18(3), 254–260. 10.1111/j.1467-9280.2007.01885.x, 17444923 PMC3864753

[bib34] Hay, J. F., Pelucchi, B., Graf Estes, K., & Saffran, J. R. (2011). Linking sounds to meanings: Infant statistical learning in a natural language. Cognitive Psychology, 63(2), 93–106. 10.1016/j.cogpsych.2011.06.002, 21762650 PMC3143199

[bib35] Hay, J. F., & Saffran, J. R. (2012). Rhythmic grouping biases constrain infant statistical learning. Infancy, 17(6), 610–641. 10.1111/j.1532-7078.2011.00110.x, 23730217 PMC3667627

[bib36] Horst, J. S., & Hout, M. C. (2016). The Novel Object and Unusual Name (NOUN) Database: A collection of novel images for use in experimental research. Behavior Research Methods, 48(4), 1393–1409. 10.3758/s13428-015-0647-3, 26424438

[bib37] Johnson, E. K. (2016). Constructing a proto-lexicon: An integrative view of infant language development. Annual Review of Linguistics, 2, 391–412. 10.1146/annurev-linguistics-011415-040616

[bib38] Johnson, E. K., Seidl, A., & Tyler, M. D. (2014). The edge factor in early word segmentation: Utterance-level prosody enables word form extraction by 6-month-olds. PLoS ONE, 9(1), Article e83546, 10.1371/journal.pone.0083546, 24421892 PMC3885442

[bib39] Johnson, E. K., & Tyler, M. D. (2010). Testing the limits of statistical learning for word segmentation. Developmental Science 13(2), 339–345. 10.1111/j.1467-7687.2009.00886.x, 20136930 PMC2819668

[bib40] Jones, B. K., Johnson, M., & Frank, M. C. (2010). Learning words and their meanings from unsegmented child-directed speech. In Human Language Technologies: The Annual Conference of the North American Chapter of the Association for Computational Linguistics (pp. 501–509). Association for Computational Linguistics. https://aclanthology.org/N10-1074/

[bib41] Karaman, F., & Hay, J. F. (2018). The longevity of statistical learning: When infant memory decays, isolated words come to the rescue. Journal of Experimental Psychology: Learning, Memory, and Cognition, 44(2), 221–232. 10.1037/xlm0000448, 28782968 PMC5803482

[bib42] Krogh, L., Vlach, H. A., & Johnson, S. P. (2013). Statistical learning across development: Flexible yet constrained. Frontiers in Psychology, 3, Article 598. 10.3389/fpsyg.2012.00598, 23430452 PMC3576810

[bib43] Lany, J., & Saffran, J. R. (2010). From statistics to meaning: Infants’ acquisition of lexical categories. Psychological Science, 21(2), 284–291. 10.1177/0956797609358570, 20424058 PMC3865606

[bib44] Mattys, S. L., & Jusczyk, P. W. (2001). Do infants segment words or recurring contiguous patterns? Journal of Experimental Psychology: Human Perception and Performance, 27(3), 644–655. 10.1037/0096-1523.27.3.644, 11424651

[bib45] Mattys, S. L., Jusczyk, P. W., Luce, P. A., & Morgan, J. L. (1999). Phonotactic and prosodic effects on word segmentation in infants. Cognitive Psychology, 38(4), 465–494. 10.1006/cogp.1999.0721, 10334878

[bib46] Mersad, K., & Nazzi, T. (2011). Transitional probabilities and positional frequency phonotactics in a hierarchical model of speech segmentation. Memory & Cognition, 39(6), 1085–1093. 10.3758/s13421-011-0074-3, 21312017

[bib47] Mirman, D., Magnuson, J. S., Graf Estes, K., & Dixon, J. A. (2008). The link between statistical segmentation and word learning in adults. Cognition, 108(1), 271–280. 10.1016/j.cognition.2008.02.003, 18355803 PMC2486406

[bib48] Peirce, J., Gray, J. R., Simpson, S., MacAskill, M., Höchenberger, R., Sogo, H., Kastman, E., & Lindeløv, J. K. (2019). PsychoPy2: Experiments in behavior made easy. Behavior Research Methods, 51(1), 195–203. 10.3758/s13428-018-01193-y, 30734206 PMC6420413

[bib49] Quine, W. V. O. (1960). Word and object. MIT Press.

[bib50] R Core Team. (2021). R: A language and environment for statistical computing. R Foundation for Statistical Computing.

[bib51] Räsänen, O., & Rasilo, H. (2015). A joint model of word segmentation and meaning acquisition through cross-situational learning. Psychological Review, 122(4), 792–829. 10.1037/a0039702, 26437151

[bib52] Romberg, A. R., & Saffran, J. R. (2010). Statistical learning and language acquisition. Wiley Interdisciplinary Reviews: Cognitive Science, 1(6), 906–914. 10.1002/wcs.78, 21666883 PMC3112001

[bib53] Saffran, J. R. (2020). Statistical language learning in infancy. Child Development Perspectives, 14(1), 49–54. 10.1111/cdep.12355, 33912228 PMC8078161

[bib54] Saffran, J. R., Aslin, R. N., & Newport, E. L. (1996). Statistical learning by 8-month-old infants. Science, 274(5294), 1926–1928. 10.1126/science.274.5294.1926, 8943209

[bib55] Scheel, A. M., Tiokhin, L., Isager, P. M., & Lakens, D. (2021). Why hypothesis testers should spend less time testing hypotheses. Perspectives on Psychological Science, 16(4), 744–755. 10.1177/1745691620966795, 33326363 PMC8273364

[bib56] Shoaib, A., Wang, T., Hay, J. F., & Lany, J. (2018). Do infants learn words from statistics? Evidence from English-learning infants hearing Italian. Cognitive Science, 42(8), 3083–3099. 10.1111/cogs.12673, 30136301 PMC6589611

[bib57] Shukla, M., White, K. S., & Aslin, R. N. (2011). Prosody guides the rapid mapping of auditory word forms onto visual objects in 6-mo-old infants. Proceedings of the National Academy of Sciences of the United States of America, 108(15), 6038–6043. 10.1073/pnas.1017617108, 21444800 PMC3076873

[bib58] Simons, D. J., Shoda, Y., & Lindsay, D. S. (2017). Constraints on Generality (COG): A proposed addition to all empirical papers. Perspectives on Psychological Science, 12(6), 1123–1128. 10.1177/1745691617708630, 28853993

[bib59] Smith, L. B, Jayaraman, S., Clerkin, E., & Yu, C. (2018). The developing infant creates a curriculum for statistical learning. Trends in Cognitive Sciences, 22(4), 325–336. 10.1016/j.tics.2018.02.004, 29519675 PMC5866780

[bib60] Smith, L. B., Suanda, S. H., & Yu, C. (2014). The unrealized promise of infant statistical word-referent learning. Trends in Cognitive Sciences, 18(5), 251–258. 10.1016/j.tics.2014.02.007, 24637154 PMC4009695

[bib61] Smith, L. B., & Yu, C. (2008). Infants rapidly learn word-referent mappings via cross-situational statistics. Cognition, 106(3), 1558–1568. 10.1016/j.cognition.2007.06.010, 17692305 PMC2271000

[bib62] Smith, L. B., & Yu, C. (2013). Visual attention is not enough: Individual differences in statistical word-referent learning in infants. Language Learning and Development, 9(1), 25–49. 10.1080/15475441.2012.707104, 24403867 PMC3882028

[bib100] Steber, S., & Rossi, S. (2020). So young, yet so mature? Electrophysiological and vascular correlates of phonotactic processing in 18-month-olds. Developmental Cognitive Neuroscience, 43, Article 100784. 10.1016/j.dcn.2020.100784, 32510350 PMC7184260

[bib63] Storkel, H. L., Bontempo, D. E., Aschenbrenner, A. J., Maekawa, J., & Lee, S.-Y. (2013). The effect of incremental changes in phonotactic probability and neighborhood density on word learning by preschool children. Journal of Speech, Language, and Hearing Research, 56(5), 1689–1700. 10.1044/1092-4388(2013/12-0245), 23882005 PMC4049320

[bib64] Sundara, M., Zhou, Z. L., Breiss, C., Katsuda, H., & Steffman, J. (2022). Infants’ developing sensitivity to native language phonotactics: A meta-analysis. Cognition, 221, Article 104993. 10.1016/j.cognition.2021.104993, 34953268

[bib65] Swingley, D. (1999). Conditional probability and word discovery: A corpus analysis of speech to infants. In M. Hahn & S. C. Stoness (Eds.), Proceedings of the 21st Annual Conference of the Cognitive Science Society (pp. 724–729). Psychology Press. 10.4324/9781410603494-131

[bib66] Tachakourt, Y. (2023). Simultaneous speech segmentation and cross-situational statistical learning in monolinguals, bilinguals, and multilinguals. Journal of Applied Language and Cultural Studies, 6(1), 110–134. https://revues.imist.ma/index.php/JALCS/article/view/36374/18533

[bib67] Thiessen, E. D. (2010). Effects of visual information on adults’ and infants’ auditory statistical learning. Cognitive Science, 34(6), 1093–1106. 10.1111/j.1551-6709.2010.01118.x, 21564244

[bib68] Vitevitch, M. S., & Luce, P. A. (2004). A Web-based interface to calculate phonotactic probability for words and nonwords in English. Behavior Research Methods, Instruments, & Computers, 36(3), 481–487. 10.3758/BF03195594, 15641436 PMC2553700

[bib69] Vlach, H. A., & DeBrock, C. A. (2017). Remember dax? Relations between children’s cross-situational word learning, memory, and language abilities. Journal of Memory and Language, 93, 217–230. 10.1016/j.jml.2016.10.001, 28503024 PMC5425170

[bib70] Vlach, H. A., & Johnson, S. P. (2013). Memory constraints on infants’ cross-situational statistical learning. Cognition, 127(3), 375–382. 10.1016/j.cognition.2013.02.015, 23545387 PMC4099971

[bib71] Yang, C. D. (2004). Universal Grammar, statistics or both? Trends in Cognitive Sciences, 8(10), 451–456. 10.1016/j.tics.2004.08.006, 15450509

[bib72] Yu, C., & Smith, L. B. (2007). Rapid word learning under uncertainty via cross-situational statistics. Psychological Science, 18(5), 414–420. 10.1111/j.1467-9280.2007.01915.x, 17576281

[bib73] Yu, C., Zhang, Y., Slone, L. K., & Smith, L. B. (2021). The infant’s view redefines the problem of referential uncertainty in early word learning. Proceedings of the National Academy of Sciences of the United States of America, 118(52), Article e2107019118. 10.1073/pnas.2107019118, 34933998 PMC8719889

[bib74] Yurovsky, D., & Frank, M. C. (2015). An integrative account of constraints on cross-situational learning. Cognition, 145, 53–62. 10.1016/j.cognition.2015.07.013, 26302052 PMC4661069

[bib75] Yurovsky, D., Yu, C., & Smith, L. B. (2012). Statistical speech segmentation and word learning in parallel: Scaffolding from child-directed speech. Frontiers in Psychology, 3, Article 374. 10.3389/fpsyg.2012.00374, 23162487 PMC3498894

[bib76] Yurovsky, D., Yu, C., & Smith, L. B. (2013). Competitive processes in cross-situational word learning. Cognitive Science, 37(5), 891–921. 10.1111/cogs.12035, 23607610 PMC3701745

